# Supplementary material for: Estimation of Respiratory Syncytial Virus-attributable hospitalizations among older adults in Japan between 2015 and 2018: An administrative health claims database analysis
Source: PLoS One. 2026 Mar 17;21(3):e0344294. doi: 10.1371/journal.pone.0344294 (PMC12994811; doi:10.1371/journal.pone.0344294)
Supplement: S1 Table — (DOCX) [file pone.0344294.s001.docx]

S1 Table. Definition of hospitalization main and secondary outcomes

| **Outcome** | **ICD-10 code^†^** |
| --- | --- |
| **Cardiorespiratory diseases** | J00-J99, I21, I48, I49, I50, I63, I64 |
| **Respiratory diseases** | J00-J99 |
| **Influenza or pneumonia** | J09-18 |
| **Chronic lower respiratory disease** | J40-47 |
| **Chronic heart failure exacerbation** | I42-43; I50; I51.7 |

† International Statistical Classification of Diseases and Related Health Problems 10th Revision (ICD-10)-WHO, Version 2015. [https://icd.who.int/browse10/2015/en#/](https://icd.who.int/browse10/2010/en#/) Note: In Japan, ICD-10 coding has been in use since 1990.
