# Supplementary material for: Estimation of Respiratory Syncytial Virus-attributable hospitalizations among older adults in Japan between 2015 and 2018: An administrative health claims database analysis
Source: PLoS One. 2026 Mar 17;21(3):e0344294. doi: 10.1371/journal.pone.0344294 (PMC12994811; doi:10.1371/journal.pone.0344294)
Supplement: S2 Table — (DOCX) [file pone.0344294.s002.docx]

S2 Table. Annual number of hospitalizations due to cardiorespiratory diseases (based on the source data [non-extrapolated]) by age group, January 2015–June 2019, Japan

| **Year** | **60–79 years** | **≥80 years** |
| --- | --- | --- |
| **Cardiorespiratory diseases** | | |
| 2015 | 84,732 | 97,227 |
| 2016 | 99,817 | 116,565 |
| 2017 | 117,904 | 141,718 |
| 2018 | 132,603 | 159,883 |
| 2019^†^ | 74,344 | 91,664 |
| **Respiratory diseases** | | |
| 2015 | 42,021 | 55,466 |
| 2016 | 49,495 | 65,160 |
| 2017 | 56,546 | 78,874 |
| 2018 | 63,074 | 88,252 |
| 2019^†^ | 35,718 | 50,524 |
| **Influenza or pneumonia** | | |
| 2015 | 17,107 | 23,897 |
| 2016 | 20,771 | 28,583 |
| 2017 | 23,509 | 35,546 |
| 2018 | 27,092 | 40,858 |
| 2019^†^ | 16,086 | 24,983 |
| **Chronic lower respiratory disease** | | |
| 2015 | 4,244 | 3,593 |
| 2016 | 5,001 | 4,312 |
| 2017 | 6,092 | 5,296 |
| 2018 | 6,604 | 5,902 |
| 2019^†^ | 3,742 | 3,317 |
| **Chronic heart failure exacerbation** | | |
| 2015 | 14,041 | 22,830 |
| 2016 | 15,728 | 28,397 |
| 2017 | 19,591 | 35,789 |
| 2018 | 21,997 | 40,620 |
| 2019^†^ | 12,741 | 24,184 |

† The year 2019 has incomplete data (until 30 June 2019)
