# Supplementary material for: Estimation of Respiratory Syncytial Virus-attributable hospitalizations among older adults in Japan between 2015 and 2018: An administrative health claims database analysis
Source: PLoS One. 2026 Mar 17;21(3):e0344294. doi: 10.1371/journal.pone.0344294 (PMC12994811; doi:10.1371/journal.pone.0344294)
Supplement: S3 Table — (DOCX) [file pone.0344294.s003.docx]

**S3 Table.** **Annual number of RSV and influenza proxy DPC (Diagnosis Procedure Combination) hospitalizations (based on the source data [non-projected]), January 2015–June 2019, Japan**

| **Year** | **Number of RSV proxy cases** | **Number of influenza proxy cases** |
| --- | --- | --- |
| 2015 | 35,288 | 6,928 |
| 2016 | 33,008 | 8,064 |
| 2017 | 46,452 | 14,388 |
| 2018 | 45,652 | 17,780 |
| 2019^†^ | 13,992 | 17,160 |

† The year 2019 has incomplete data (until 30 June 2019)
