# Supplementary material for: Estimation of Respiratory Syncytial Virus-attributable hospitalizations among older adults in Japan between 2015 and 2018: An administrative health claims database analysis
Source: PLoS One. 2026 Mar 17;21(3):e0344294. doi: 10.1371/journal.pone.0344294 (PMC12994811; doi:10.1371/journal.pone.0344294)

**S1 Fig. Weekly number of RSV and influenza proxy DPC hospitalizations (based on the source data [non-projected]), January 2015–June 2019, Japan**


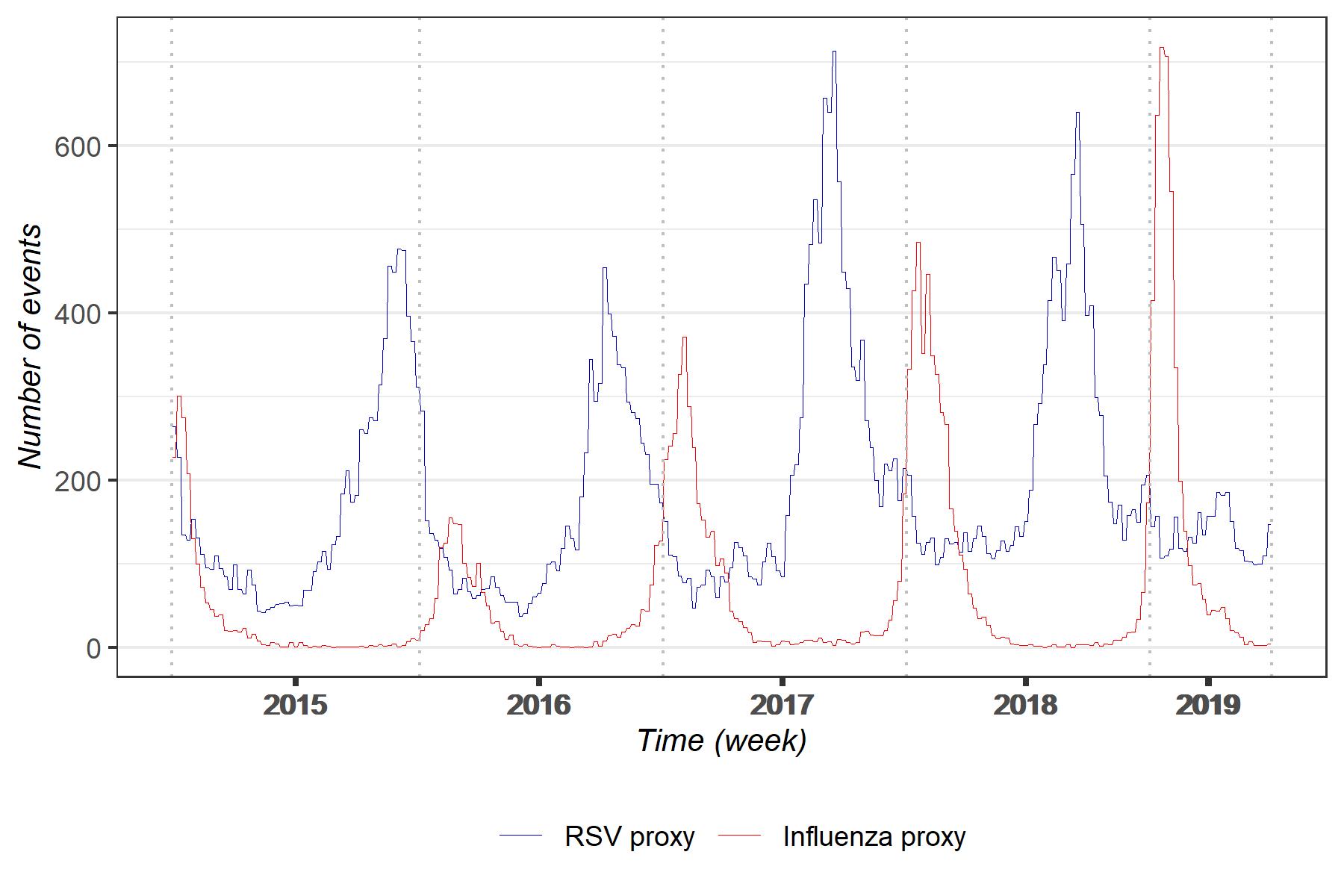

Supplement: S1 Fig — (DOCX) [file pone.0344294.s004.docx]
