# Supplementary material for: Estimation of Respiratory Syncytial Virus-attributable hospitalizations among older adults in Japan between 2015 and 2018: An administrative health claims database analysis
Source: PLoS One. 2026 Mar 17;21(3):e0344294. doi: 10.1371/journal.pone.0344294 (PMC12994811; doi:10.1371/journal.pone.0344294)
Supplement: Attachment — Submitted filename: report_10.1101+2025.01.21.25320883.pdf [file pone.0344294.s005.pdf]

ScreenIT Report

This is an automated report for 10.1101+2025.01.21.25320883.pdf. This report was solicited by the PLOS One editorial team and provided by ScreenIT.

ScreenIT is an independent group of scientists developing automated tools that analyze academic papers. A set of automated tools screened your submitted manuscript and provided the report below. Each tool was created by your academic colleagues with the goal of helping authors.

The tools look for factors that are important for transparency, rigor and reproducibility, and we hope that the report might help you to improve reporting in your manuscript. Within the report you will find links to more information about the items that the tools check. These links include helpful papers, websites, or videos that explain why the item is important.

While our screening tools aim to improve and maintain quality standards they may, on occasion, miss nuances specific to your study type or flag something incorrectly. Each tool has limitations that are described on the ScreenIT website. The tools screen the main file for the paper; they are not able to screen supplements stored in separate files.

Please note that the Academic Editor had access to these comments while making a decision on your manuscript. The Academic Editor may ask that issues flagged in this report be addressed.

If you would like to provide feedback on the ScreenIT tool, please email the team at ScreenIt@bih-charite.de. If you have questions or concerns about the review process, please contact the PLOS One office at plosone@plos.org.

How to read the report:

- no action required

? - review for potential issues

! - a critical issue was found by a tool

n/a - may not be applicable to your paper

- more information about this item can be obtained using this link

Rigor

n/a	Ethics	not required.
✓	Inclusion and Exclusion Criteria	*"Records that had the same admission and discharge date were excluded."*
✓	Flow charts and attrition	Thank you for including a study flow chart and information about attrition and exclusions to help readers evaluate the risk of bias.<br><br>Sentence about attrition: *"not required."*
?	Sex as a biological variable	Not detected. Please consider reporting the sex of human participants, animals or cells, if you haven't done so.
✓	Subject Demographics	Age: *"The IRs of RSV-attributable hospitalizations for individuals aged 60 years and older were obtained by summing the projected yearly model-based numbers of RSV-attributable hospitalizations for 6079 years, and 80 years and older."*
?	Randomization	Not detected. If you performed an experimental study, please specify whether participants, animals or samples were randomly assigned to treatments or groups, and specify the randomization procedure.
?	Blinding	Not detected. Please specify whether blinding was used at various phases of the experiment (e.g., blinding of patients, caregivers, outcome assessments, data analysis).
?	Power Analysis	Not detected. Please state how you determined the sample size for the study. If you performed a power or sample size calculation, please provide details.
n/a	Replication	not required.

Generated by [Barzooka (flow charts)](), [SciScore (everything else)]().
SciScore only screens the text of the methods section; we apologize if the tool has missed items reported elsewhere in the paper or supplements.

Resources

No key resources detected.
Generated by SciScore. SciScore only screens the text of the methods section; we apologize if the tool has missed items reported elsewhere in the paper or supplements.

Transparency

?	Open code	If you wrote code to analyze your data, please consider sharing the code in a public repository. This makes it easier for others to reproduce your analyses, and may also aid others seeking to analyze similar datasets.
?	Open data	If permitted, sharing data on a public repository with a persistent identifier (a DOI or accession number) can improve reproducibility and make it easier for other scientists to expand on your work. Papers with open data are cited more often than papers without open data. Some institutions have an expert who can provide advice on data sharing.
✓	Self-acknowledged limitations	*"Our study had several limitations. First, all Japan regions were pooled into one dataset for modeling, while different regions are likely to display different RSV seasonality patterns (e.g., Okinawa region has a subtropical climate [30]). Unfortunately, we were not able to account for this by stratifying by region as no appropriate geographical indicator was available in the source data. We also did not include proxies for the activity of other respiratory pathogens in our model, which might have resulted in an overestimation of RSV burden. However, even without explicitly modeling other potentially relevant pathogens, they are largely accounted for in the model indirectly through the periodic component and the overdispersion parameter. Lastly, our projections relied heavily on several assumptions that could not be verified concerning the consistency of the distribution of RSV burden across the different hospital types (MDV/non-MDV DPC hospitals, DPC/non-DPC hospitals) and age groups (..."*
✓	Conflict of interest statement	Thank you for stating any conflicts of interest.

✓	Funding statement	Thank you for reporting your funding source(s), or stating that there was no specific funding for this work.
✓	Registration statement	Thank you for registering your study.

Generated by ODDPub, limitation-recognizer, rtransparent, trial-identifier.

Figures

✓	No bar graphs of continuous data	Thank you for not using bar graphs to present continuous data. Many different datasets and data distributions can lead to the same bar graph, and the actual data may suggest different conclusions from the summary statistics alone.
✓	No rainbow color maps	Thank you for not using rainbow colormaps. Rainbow color maps distort readers' perception of the data by introducing visual artifacts and are not accessible to colorblind readers.

Generated by JetFighter, Barzooka.
