# Supplementary material for: Estimation of Respiratory Syncytial Virus-attributable hospitalizations among older adults in Japan between 2015 and 2018: An administrative health claims database analysis
Source: PLoS One. 2026 Mar 17;21(3):e0344294. doi: 10.1371/journal.pone.0344294 (PMC12994811; doi:10.1371/journal.pone.0344294)
Supplement: Attachment — Submitted filename: Response_to_Editorial_Comments_Data_availability_RSV_Japan.docx [file pone.0344294.s007.docx]

Dr. Liling Chaw

Academic Editor

PLOS ONE

Dear Dr. Chaw,

We are grateful for the opportunity to revise the manuscript in light of the reviewers’ comments and journal requirements. We have responded to all comments as itemized in our point-by-point reply below in blue. All previous changes (previous revision requested on Friday, July 25, 2025) are saved in tracked changes in the revised version (file name with ending: Manuscript_with_Track_Changes) of our manuscript. An unmarked version of your revised paper without tracked changes (file name with ending: Manuscript).

Sincerely yours,

Caihua Liang

on behalf of the authors

**Revision requested on Wednesday, September 17, 2025**

**Changes to comply with the Data Policy of PLOS ONE**

1. We understand that your data availability statement currently reads as follows: "The datasets generated and/or analyzed during the current study are not publicly available. The data are owned by Medical Data Vision (MDV) Co., Ltd, and the authors do not have permission to share the data. The data are available upon request to MDV (<https://en.mdv.co.jp>). The statistical codes are available upon request to the corresponding author.”

Please be aware, regarding your statistical codes, that PLOS’ Data Policy states that authors cannot be the sole named individuals responsible for ensuring data access.

In light of this, please respond to the following prompts:

a.) If there are no ethical or legal restrictions, please upload the minimal anonymized data set necessary to replicate your study findings to a stable, public repository and provide us with the relevant URLs, DOIs, or accession numbers. You also have the option of uploading the data as Supporting Information files, but we would recommend depositing data directly to a data repository if possible.

*Response: Minimal data for this study can not be shared publicly, because the data was obtained from a third-party (Medical Data Vision).*

b.) If there are ethical or legal restrictions on sharing a de-identified data set, please explain them in detail (e.g., data contain potentially identifying or sensitive patient information, data are owned by a third-party organization, etc.) and who has imposed them (e.g., a Research Ethics Committee or Institutional Review Board, etc.). Please also provide non-author contact information for a data access committee, ethics committee, or other institutional body to which data requests may be sent.

*Response: Minimal data for this study can not be shared publicly, because the data was obtained from a third-party (Medical Data Vision). The Data Availability Statement has been revised to include details regarding data access restrictions and information about the data owner.*

*Updated Data Availability Statement:*

*“The datasets generated during and/or analyzed during the current study are not publicly available. The data are owned by* *a third-party, Medical Data Vision (MDV) Co., Ltd, and the authors do not have permission to share the data. The data are available upon request to MDV (*[*https://en.mdv.co.jp*](https://en.mdv.co.jp); *contacts: Hiroyuki Tada [email:* [*tada@mdv.co.jp*](mailto:tada@mdv.co.jp)*] and Yuki Santo [email:* [*santo_yuki@mdv.co.jp*](mailto:santo_yuki@mdv.co.jp)*]).*

*The statistical codes are available upon request to the following contacts: statistics department, P95 (email:* [*stat@p-95.com*](mailto:stat@p-95.com)*), and Hannah Volkman (Pfizer Inc; email:* [*Hannah.Volkman@pfizer.com*](mailto:Hannah.Volkman@pfizer.com)*).”*
