# Supplementary material for: Estimation of Respiratory Syncytial Virus-attributable hospitalizations among older adults in Japan between 2015 and 2018: An administrative health claims database analysis
Source: PLoS One. 2026 Mar 17;21(3):e0344294. doi: 10.1371/journal.pone.0344294 (PMC12994811; doi:10.1371/journal.pone.0344294)
Supplement: Attachment — Submitted filename: Response_to_Reviewers_RSV_Japan_revision3_PLOS_One.docx [file pone.0344294.s008.docx]

Dr. Liling Chaw

Academic Editor

PLOS ONE

Date: 08 January 2026

Dear Dr. Chaw,

We are grateful for the opportunity to revise the manuscript in light of the reviewers’ comments and journal requirements. We have responded to all comments as itemized in our point-by-point reply below in blue. All changes are saved in tracked changes in the revised version (file name with: Revised_Manuscript_with_Track_Changes) of our manuscript. An unmarked version of our revised manuscript without tracked changes (file name with: Manuscript) is also shared.

Sincerely yours,

Caihua Liang

on behalf of the authors

**Journal Requirements:**

If the reviewer comments include a recommendation to cite specific previously published works, please review and evaluate these publications to determine whether they are relevant and should be cited. There is no requirement to cite these works unless the editor has indicated otherwise.

*Response: Not applicable. The inclusion of the newly cited publications (Refs. 21, 24-27, 35-39, 41, 42) was necessary for the updated version of the manuscript.*

Please review your reference list to ensure that it is complete and correct. If you have cited papers that have been retracted, please include the rationale for doing so in the manuscript text, or remove these references and replace them with relevant current references. Any changes to the reference list should be mentioned in the rebuttal letter that accompanies your revised manuscript. If you need to cite a retracted article, indicate the article’s retracted status in the References list and also include a citation and full reference for the retraction notice.

*Response: Not applicable. This has been reviewed and verified.*

**Reviewers' comments:**

Reviewer's Responses to Questions

**Comments to the Author**

1. If the authors have adequately addressed your comments raised in a previous round of review and you feel that this manuscript is now acceptable for publication, you may indicate that here to bypass the “Comments to the Author” section, enter your conflict of interest statement in the “Confidential to Editor” section, and submit your "Accept" recommendation.

Reviewer #2: All comments have been addressed

Reviewer #3: (No Response)

*Response: We thank reviewer #2 for the confirmation.*

2. Is the manuscript technically sound, and do the data support the conclusions?

The manuscript must describe a technically sound piece of scientific research with data that supports the conclusions. Experiments must have been conducted rigorously, with appropriate controls, replication, and sample sizes. The conclusions must be drawn appropriately based on the data presented.

Reviewer #2: Yes

Reviewer #3: Partly

*Response: We thank the reviewers for their (partial) confirmation. We have addressed the concerns raised by Reviewer #3; please find our detailed answers under section 6.*

3. Has the statistical analysis been performed appropriately and rigorously?

Reviewer #2: Yes

Reviewer #3: Yes

*Response: We thank the reviewers for their confirmation.*

4. Have the authors made all data underlying the findings in their manuscript fully available?

The [PLOS Data policy](https://eur02.safelinks.protection.outlook.com/?url=https%3A%2F%2Furldefense.com%2Fv3%2F__http%3A%2Ftrack.editorialmanager.com%2FCL0%2Fhttp%3A*2F*2Fwww.plosone.org*2Fstatic*2Fpolicies.action*23sharing%2F1%2F010f019ae1c1f3d6-8d7950b4-7723-4c72-9624-578f8e1cb2ad-000000%2FAREOSS-9jWql65hulmA_UrGbPWo4-1eSj2R8eUlMj84%3D240__%3BJSUlJSU!!H9nueQsQ!6lnccrBD8b-BWlahv9YLlFRCR3M_6JfSy7AVomD1QycchHIegwsoRQFrsHJ56_MMPVRovxKjgdc_2vMxoso%24&data=05%7C02%7Csomsuvro.basu%40p-95.com%7C764110fb758244393b7608de3220f1a6%7Ca3ca6855f73146e09a48a088d208b05e%7C1%7C0%7C639003314245054912%7CUnknown%7CTWFpbGZsb3d8eyJFbXB0eU1hcGkiOnRydWUsIlYiOiIwLjAuMDAwMCIsIlAiOiJXaW4zMiIsIkFOIjoiTWFpbCIsIldUIjoyfQ%3D%3D%7C0%7C%7C%7C&sdata=k8tjFlvAI6xvYvCjHw0XhgFR5Kqj5QngbYdbYNikTTU%3D&reserved=0) requires authors to make all data underlying the findings described in their manuscript fully available without restriction, with rare exception (please refer to the Data Availability Statement in the manuscript PDF file). The data should be provided as part of the manuscript or its supporting information, or deposited to a public repository. For example, in addition to summary statistics, the data points behind means, medians and variance measures should be available. If there are restrictions on publicly sharing data—e.g. participant privacy or use of data from a third party—those must be specified.

Reviewer #2: No

Reviewer #3: No

*Response: The Data Availability Statement was updated in the last revision round to explain why the datasets are not publicly available and how they can be requested. We share the Data Availability Statement, again below:*

***Data Availability Statement****: “The datasets generated during and/or analyzed during the current study are not publicly available. The data are owned by a third-party, Medical Data Vision (MDV) Co., Ltd, and the authors do not have permission to share the data. The data are available upon request to MDV (https://en.mdv.co.jp; contacts: Hiroyuki Tada [email: tada@mdv.co.jp] and Yuki Santo [email: santo_yuki@mdv.co.jp]).*

*The statistical codes are available upon request to the following contacts: statistics department, P95 (email: stat@p-95.com), and Hannah Volkman (Pfizer Inc; email: Hannah.Volkman@pfizer.com).”*

5. Is the manuscript presented in an intelligible fashion and written in standard English?

PLOS ONE does not copyedit accepted manuscripts, so the language in submitted articles must be clear, correct, and unambiguous. Any typographical or grammatical errors should be corrected at revision, so please note any specific errors here.

Reviewer #2: Yes

Reviewer #3: Yes

*Response: We thank the reviewers for their confirmation.*

6. Review Comments to the Author

Please use the space provided to explain your answers to the questions above. You may also include additional comments for the author, including concerns about dual publication, research ethics, or publication ethics. (Please upload your review as an attachment if it exceeds 20,000 characters)

**Reviewer #2:**

The authors have addressed the comments raised in the methodology by naming the time series model that was used. They have also added a table of the descriptives too.

*Response: Thank you for taking the time to review our work. We truly appreciate your thoughtful feedback and support.*

*-------------*

**Reviewer #3:**

I read this article on an important and timely topic. The Results present valuable numeric estimates. Significant revisions (some of which have already been mentioned in the discussion) are required to clarify the methodology, justify key assumptions, and validate data sources. Contextualization relative to national level is insufficient. I recommend major revision.

**Introduction**

- L74: If available, report the actual proportion of adults aged 60+ years in Japan instead of stating “a large proportion.”

*Response: Thank you for your comment. We have added the following information: “…**representing 41% in 2019 [14]” (line 75 in the clean revised manuscript).*

- L75: specify which new vaccines are available for adults aged +60 years.

*Response: We have added the following information: “..**such as the protein subunit vaccines and mRNA vaccine,” (lines 76-77 in the clean revised manuscript).*

**Methods**

- L114-117: Some important cardiovascular categories are not included.

*Response:* *As described in our generic protocol (1), we have included the selected cardiovascular disease codes most likely to be associated with RSV, as previously reported in the literature (2-9) and used in other model-based studies.*

1) *Bruyndonckx R, Polkowska-Kramek A, Liang C, Nuttens C, Tran TMP, Gessner BD, et al. Estimation of symptomatic respiratory syncytial virus infection incidence in adults in multiple countries: a time-series model-based analysis protocol. Infectious Diseases and Therapy. 2024;13(4):953-63. doi: 10.1007/s40121-024-00948-9.*
2) *Kujawski SA, Whitaker M, Ritchey MD, Reingold AL, Chai SJ, Anderson EJ, et al. Rates of respiratory syncytial virus (RSV)-associated hospitalization among adults with congestive heart failure-United States, 2015–2017. PLoS ONE. 2022;17(3): e0264890.*
3) *Ivey KS, Edwards KM, Talbot HK. Respiratory syncytial virus and associations with cardio‑vascular disease in adults. J Am Coll Cardiol. 2018;71(14):1574–83.*
4) *Franczuk P, Tkaczyszyn M, Kulak M, Domenico E,Ponikowski P, Jankowska EA. Cardiovascular complications of viral respiratory infections and COVID-19. Biomedicines. 2022.* [*https://doi.org/10.3390/biomedicines11010071*](https://doi.org/10.3390/biomedicines11010071)*.*
5) *Zhou H, Thompson WW, Viboud CG, Ringholz CM, Cheng PY, Steiner C, et al. Hospitalizations associated with influenza and respiratory syncytial virus in the United States, 1993–2008. Clin Infect Dis. 2012;54(10):1427–36*
6) *Matias G, Taylor RJ, Haguinet F, Schuck-Paim C, Lustig RL, Shinde V. Estimates of mortality attributable to influenza and RSV in the United States during 1997–2009 by influenza type or subtype, age, cause of death, and risk status. Influenza Other Respir Viruses. 2014;8(5):507–15*
7) *Mullooly J, Bridges C, Thompson W, Chen J, Weintraub E, Jackson L, et al. Influenza- and RSV-associated hospitalizations among adults. Vaccine.2007;25(5):846–55.*
8) *Kawashima H, Inagaki N, Nakayama T, Morichi S, Nishimata S, Yamanaka G, et al. Cardiac complications caused by respiratory syncytial virus infection: questionnaire survey and a literature review. Glob Pediatr Health. 2021.* [*https://doi.org/10.1177/*](https://doi.org/10.1177/) *2333794X211044114*
9) *Corrales-Medina VF, Madjid M, Musher DM. Role of acute infection in triggering acute coronary syndromes. Lancet Infect Dis. 2010;10(2):83–92.*

*We have added this information (lines 114-115* *in the clean revised manuscript): “As described in our generic protocol [8], we have included the selected cardiovascular disease codes most likely to be associated with RSV*.”

- L124-128: The choice of J21.9 as part of the RSV proxy for children <2 is not supported by Japanese validation data. The RSV proportion for this population category has been shown to be around 60% and is highly variable (doi: 10.1371/journal.pone.0242302).

*Response: The more generic bronchiolitis code (J21.9 or 466.1) is included in the RSV proxy to accommodate for the reduction in RSV testing during the peak and tail of the season, which we have observed in administrative databases in several countries. Additionally, it serves as an indicator marking the start of the RSV season (1-5). The proxies seek to accurately track the relative level of viral activity in the community, so the absolute value of the activity is less important than consistent measurement across the year to accurately track relative trends (Cf. generic protocol).*

1) *Calvo C, Pozo F, García-García ML, Sanchez M, Lopez-Valero M, Pérez-Breña P, et al. Detection of new respiratory viruses in hospitalized infants with bronchiolitis: a three-year prospective study. Acta Paediatr. 2010;99(6):883–887. doi: 10.1111/j.1651-2227.2010.01714.x.*
2) *Bandeira T, Carmo M, Lopes H, Gomes C, Martins M, Guzman C, et al. Burden and severity of children's hospitalizations by respiratory syncytial virus in Portugal, 2015–2018. Influenza Other Respir Viruses. 2023;17(1):e13066. doi: 10.1111/irv.13066.*
3) *Mansbach JM, Piedra PA, Teach SJ, Sullivan AF, Forgey T, Clark S, et al. Prospective multicenter study of viral etiology and hospital length of stay in children with severe bronchiolitis. Arch Pediatr Adolesc Med. 2012;166(8):700–706. doi: 10.1001/archpediatrics.2011.1669.*
4) *Walton NA, Poynton MR, Gesteland PH, Maloney C, Staes C, Facelli JC. Predicting the start week of respiratory syncytial virus outbreaks using real time weather variables. BMC Med Inform Decis Mak. 2010;10:68. doi: 10.1186/1472-6947-10-68.*
5) *Kenmoe S, Kengne-Nde C, Ebogo-Belobo JT, Mbaga DS, Fatawou Modiyinji A, Njouom R. Systematic review and meta-analysis of the prevalence of common respiratory viruses in children < 2 years with bronchiolitis in the pre-COVID-19 pandemic era. PLoS One. 2020;15(11):e0242302. doi: 10.1371/journal.pone.0242302.*

*The following sentence is slightly modified, adding the above references and information (lines 130-133 in the clean revised manuscript): “We included acute bronchiolitis, unspecified (J21.9), because RSV is responsible for the majority of bronchiolitis hospitalizations in this age group [23, 24] and was found to be a good indicator for the start of the RSV season [23-27].”*

- L128-129: The influenza proxy is based on hospitalizations in adults +65 years, although the study population is +60 years.

*Response: Thank you very much for flagging this. This was a typo, and we have replaced (line 134 in the clean revised manuscript) 65 years by 60 years.*

- The manuscript does not describe the source, reliability, or testing practices underlying RSV and Influenza proxies.

*Response: Please see lines 124-126 (in the clean revised manuscript): “As described in the generic protocol [8], we accounted for the circulation of selected pathogens (RSV and influenza) using hospital-based viral proxies for community-wide viral activity derived from MDV.” The underlying testing practices in each of the participated hospitals in MDV database are unknown. However, in general, hospital-based proxies have been found to be better predictors of RSV activity than surveillance data.*

*We have added a sentence, “**The latter have been shown to better predict hospitalization than surveillance-based proxies [21].” (line 126 in the clean revised manuscript).*

- L179-184: Excluding an entire year because data are incomplete could be suboptimal. A sensitivity analysis including partial 2019 data could strengthen the robustness of the results.
  The 2015-2018 window (4 years) is relatively short.

*Response: Please see lines 188-191 (in the clean revised manuscript) for the explanation that we have slightly modified by replacing “take into account” by “report”: “We modeled the hospitalization data until 30th June 2019 to make use of the most recent available data (prior to the COVID-19 pandemic). However, we did not report the results for the year 2019 since the data are incomplete….”*

- L153-155: The scaling factor assumes DPC and non-DPC hospitals are comparable, which is unlikely (differences in patient age, specialization…).
- L.171-174: The manuscript does not discuss known structural differences between DPC and non-DPC hospitals (e.g., geriatric, rehabilitation facilities), which could significantly affect RSV burden estimates.

*Response: Please note that the scaling factor described in lines 157-160 (in the clean revised manuscript) was used to project the estimated number of RSV-attributable hospitalizations to all DPC hospitals in Japan using a scaling factor corresponding to the number of all DPC hospitals in Japan divided by the number of DPC hospitals in the MDV database. We then carried out projections to all hospitals (including non-DPC hospitals) using two different types of scaling factor and acknowledging the uncertainty related to these projections and provided estimate ranges (please see lines 169-183, 258-272, 342-346 in the clean revised manuscript).*

*To clarify further, we modified the following sentences (lines 170-178 in the clean revised manuscript): “To project the IRs of RSV-attributable DPC hospitals to all hospitals,* *while accounting for the uncertainty linked to structural differences between DPC and non-DPC hospitals, we carried out* *two different projections by multiplying the estimated IRs of RSV-attributable DPC hospitals by different sets of year-specific scaling factors. The first scaling factor is defined as the total number of beds in all hospitals divided by the total number of beds in the DPC hospitals [25]. The second scaling factor was calculated by comparing the outcome-specific hospitalizations obtained from the MDV database to those from the JMDC Inc. payer-based database, which covers all hospital types in Japan and is representative of working adults younger than 65 years [27].”*

- L168-170: The second scaling method, using adults aged 50-59 years, relies on an unproven assumption that patterns in 50-59 years reflect those +60. This needs justification.

*Response: Thank you for raising this important point. According to our feasibility analysis, there is no single hospitalization data source available for Japan which accurately represents all adult age groups and all types of hospitals (DPC and non-DPC hospitals). The JMDC payer-based database is representative of all hospitalizations among working adults younger than 65 years of age, but contains limited information for people 65-74 years of age, and does not cover people >75 years of age. Therefore, we have considered for our scaling factor the JMDC age category 50-59 years, which was the most representative while being as close as possible to the age distribution of our study population.*

*We modified the following sentences (lines 175-180 in the clean revised manuscript) to explain this further: “The second scaling factor was calculated by comparing the outcome-specific hospitalizations obtained from the MDV database to those from the JMDC Inc. payer-based database, which covers all hospital types in Japan and is representative of working adults younger than 65 years [27].* *For the purpose of deriving our scaling factor, we selected the JMDC age group 50–59 years, as it provided the most robust and representative data while closely aligning with the age distribution of our study population.”*

- L162-164: The residual bootstrap procedure lacks details (number of replicates, resampling method…).

*Response: We have added more information in lines 164-166 (in the clean revised manuscript): “Confidence intervals for IRs were obtained using residual bootstrapping with 1,000 resampled datasets generated through sampling with replacement [8].”*

- No stratification by comorbidities.

*Response*: *We did not stratify by comorbidities in our study due to the unavailability of data.*

*We have now added this to the study limitations (lines 335-337 in the clean revised manuscript): “For the same reason, we also did not stratify the burden of RSV by comorbidities, which are known to significantly influence disease severity and outcomes in older adults.”*

- No stratification by Japanese regions.

*Response: This limitation has already been acknowledged in lines 334-335: “Unfortunately, we were not able to account for this by stratifying by region, as no appropriate geographical indicator was available in the source data.”*

- No analysis of mortality.

*Response: We did not focus on mortality in our study due to the unavailability of mortality data with underlying cause of death. Instead, we analyzed hospitalizations to assess the burden of RSV among adults aged 60 years and older.*

- No data on 18-60 years.

*Response: We did not model the data for 18-60 years due to the low case counts or to the absence of clearly distinguishable seasonality. We have added this in the discussion (lines 346-349 in the clean revised manuscript): “**We did not model the data for 18-60 years due to the low case counts or to the absence of clearly distinguishable seasonality. Our study focused on individuals aged 60 years and older who are at the highest risk of severe disease, making it critical to quantify the disease burden for them to guide public health strategies.”*

- No non-RSV/Influenza proxies.

*Response: This limitation is acknowledged in lines 337-341 (in the clean revised manuscript): “We also did not include proxies for the activity of other respiratory pathogens in our model, which might have resulted in an overestimation of RSV burden. However,* *this approach has been successfully used in studies conducted in other countries [16, 17, 37, 38], and even without explicitly modeling other potentially relevant pathogens, they are largely accounted for in the model indirectly through the periodic component and the overdispersion parameter.”*

**Results**

- Lines 198-202: You report a total of 1.1 million cardiorespiratory hospitalizations in the MDV database, is it possible to contextualize these numbers in relation to national hospitalization statistics. Without such context, readers cannot evaluate whether the MDV case load is proportionally higher or lower than expected.

*Response: We have added the number found on the MHLW website in lines 207-209 (in the clean revised manuscript): “**In 2017, there were 16,222,030 new inpatients in Japan hospitals (all ages, diseases, and hospital types) [25].”*

- Although Table 2 displays year-specific incidence rates, the Results section does not discuss inter-annual fluctuations.

*Response: We have added the following statement to the Results section (lines 238-239 in the clean revised manuscript): “**The IRs remained stable across the study years, as evidenced by overlapping confidence intervals."*

- You exclude 2019 from final estimates, but the Results do not discuss whether the partial season shows deviations or supports the model’s predictive behaviour.

*Response: Thank you for your comment. We have now included a small paragraph in the Discussion section to clarify this (line 349-352 in the clean revised manuscript): “**The year 2019 was excluded from the final estimates due to incomplete data for that season, however the partial data available for 2019 did not show any deviation from the model’s predictions. Specifically, IRs for 2019 were consistent with the trends observed in the other years, as indicated by overlapping confidence intervals (data not shown).”*

**Discussion**

- Higher proportions in 60-79y and higher IRs in ≥80y could be further explained.

*Response: We have rephrased the following sentences (lines 283-288 in the clean revised manuscript): “**While the persons 60–79 years exhibited a higher proportion of RSV-attributable hospitalizations, their IRs were lower compared to those 80 years and older. This likely reflects differences in population size and competing risks. Individuals 80 years and older are more likely to be hospitalized for non-RSV reasons, which dilutes the proportion of hospitalizations specifically attributed to RSV, even if their risk of infection relative to the population size (IR) is higher.”*

- Lines 297-300: confidence intervals could be provided to strengthen the robustness of international comparisons.

*Response: We did not retrieve CIs for this information, so we modified lines 307-311 (in the clean revised manuscript) to make it more general: “RSV is now being recognized as one of the leading causes of acute respiratory infections in older adults, including among patients with pneumonia [12]. RSV pneumonia can present with imaging features that are similar to other infectious pneumonias, making it difficult to differentiate from other causes of infectious pneumonia based on radiographic imaging [30-35].”*

- Projections rely on unverifiable assumptions regarding hospital types and age groups, which may under- or overestimate burden. Validation with external datasets would have been valuable.

*Response: We have acknowledged this in the in the study limitations section and now slightly modified the following sentences (lines 342-346 in the clean revised manuscript): “Our projections relied heavily on several assumptions that could not be verified concerning the consistency of the distribution of RSV burden across the different hospital types (MDV/non-MDV DPC hospitals, DPC/non-DPC hospitals) and age groups (50-59 years/60 years and older). Given the inability to validate these assumptions with external data, we chose to present estimate ranges to reflect the associated uncertainty.”*

- Future studies should consider 18+ years, mortality...

*Response: The concluding sentence was slightly modified (lines 362-367 in the clean revised manuscript): “Future studies should include assessing vaccine-preventable burden among adults with comorbidities and against a broad group of outcomes. They should also assess non-hospitalization burden, mortality, and longer-term consequences of more severe RSV infection, such as impact on quality of life, requirements for assisted living facilities, and functioning of extended family members serving as caretakers.”*

*Please see our answer above related to studying RSV burden among individuals aged 18-60 years.*

7. PLOS authors have the option to publish the peer review history of their article ([what does this mean?](https://eur02.safelinks.protection.outlook.com/?url=https%3A%2F%2Furldefense.com%2Fv3%2F__https%3A%2Ftrack.editorialmanager.com%2FCL0%2Fhttps%3A*2F*2Fjournals.plos.org*2Fplosone*2Fs*2Feditorial-and-peer-review-process*23loc-peer-review-history%2F1%2F010f019ae1c1f3d6-8d7950b4-7723-4c72-9624-578f8e1cb2ad-000000%2FQnB09EFXJ-psz-u6tYybeUIThGHhyz5M6LlvMuVu0Kg%3D240__%3BJSUlJSUl!!H9nueQsQ!6lnccrBD8b-BWlahv9YLlFRCR3M_6JfSy7AVomD1QycchHIegwsoRQFrsHJ56_MMPVRovxKjgdc__fo1VKs%24&data=05%7C02%7Csomsuvro.basu%40p-95.com%7C764110fb758244393b7608de3220f1a6%7Ca3ca6855f73146e09a48a088d208b05e%7C1%7C0%7C639003314245077120%7CUnknown%7CTWFpbGZsb3d8eyJFbXB0eU1hcGkiOnRydWUsIlYiOiIwLjAuMDAwMCIsIlAiOiJXaW4zMiIsIkFOIjoiTWFpbCIsIldUIjoyfQ%3D%3D%7C0%7C%7C%7C&sdata=gdSyWnJz%2FHMlk5uogOXeXCC5dSE1QLXTu7WvthTpk2I%3D&reserved=0)). If published, this will include your full peer review and any attached files.

If you choose “no”, your identity will remain anonymous but your review may still be made public.

**Do you want your identity to be public for this peer review?** For information about this choice, including consent withdrawal, please see our [Privacy Policy](https://eur02.safelinks.protection.outlook.com/?url=https%3A%2F%2Furldefense.com%2Fv3%2F__https%3A%2Ftrack.editorialmanager.com%2FCL0%2Fhttps%3A*2F*2Fwww.plos.org*2Fprivacy-policy%2F1%2F010f019ae1c1f3d6-8d7950b4-7723-4c72-9624-578f8e1cb2ad-000000%2FiPyen1hLAEIzDF0nSkAirhW9YtlRUzcbtfRaZNCn0Do%3D240__%3BJSUl!!H9nueQsQ!6lnccrBD8b-BWlahv9YLlFRCR3M_6JfSy7AVomD1QycchHIegwsoRQFrsHJ56_MMPVRovxKjgdc_tUUrxYY%24&data=05%7C02%7Csomsuvro.basu%40p-95.com%7C764110fb758244393b7608de3220f1a6%7Ca3ca6855f73146e09a48a088d208b05e%7C1%7C0%7C639003314245101910%7CUnknown%7CTWFpbGZsb3d8eyJFbXB0eU1hcGkiOnRydWUsIlYiOiIwLjAuMDAwMCIsIlAiOiJXaW4zMiIsIkFOIjoiTWFpbCIsIldUIjoyfQ%3D%3D%7C0%7C%7C%7C&sdata=zD9sujq%2BRTtGX6SIUS%2FYVbOQ3BhCfDPBzPJScGc8Jzo%3D&reserved=0).

Reviewer #2: **Yes:** Oluchukwu Okorie

Reviewer #3: No
