# Supplementary material for: Estimation of Respiratory Syncytial Virus-attributable hospitalizations among older adults in Japan between 2015 and 2018: An administrative health claims database analysis
Source: PLoS One. 2026 Mar 17;21(3):e0344294. doi: 10.1371/journal.pone.0344294 (PMC12994811; doi:10.1371/journal.pone.0344294)
Supplement: Attachment — Submitted filename: Response_to_Reviewers_RSV_Japan_revision4_PLOS_One.docx [file pone.0344294.s009.docx]

Dr. Liling Chaw

Academic Editor

PLOS ONE

Date: 16 February 2026

Dear Dr. Chaw,

We are grateful for the opportunity to revise the manuscript in light of the comments from Reviewer #3. We have responded to all comments as itemized in our point-by-point reply below in blue. All changes are saved in tracked changes in the revised version (file name with: Revised_Manuscript_with_Track_Changes) of our manuscript. An unmarked version of our revised manuscript without tracked changes (file name with: Manuscript) is also shared.

Sincerely yours,

Caihua Liang

on behalf of the authors

**Reviewers' comments:**

Reviewer's Responses to Questions

**Comments to the Author**

1. If the authors have adequately addressed your comments raised in a previous round of review and you feel that this manuscript is now acceptable for publication, you may indicate that here to bypass the “Comments to the Author” section, enter your conflict of interest statement in the “Confidential to Editor” section, and submit your "Accept" recommendation.

Reviewer #3: (No Response)

*Response: We thank Reviewer #3 for the helpful feedback. We have addressed the additional concerns raised by Reviewer #3; please find our detailed answers in section 6.*

2. Is the manuscript technically sound, and do the data support the conclusions?

The manuscript must describe a technically sound piece of scientific research with data that supports the conclusions. Experiments must have been conducted rigorously, with appropriate controls, replication, and sample sizes. The conclusions must be drawn appropriately based on the data presented.

Reviewer #3: Yes

*Response: We thank Reviewer #3 for the confirmation.*

3. Has the statistical analysis been performed appropriately and rigorously?

Reviewer #3: Yes

*Response: We thank Reviewer #3 for the confirmation.*

4. Have the authors made all data underlying the findings in their manuscript fully available?

The [PLOS Data policy](https://eur02.safelinks.protection.outlook.com/?url=https%3A%2F%2Furldefense.com%2Fv3%2F__http%3A%2Ftrack.editorialmanager.com%2FCL0%2Fhttp%3A*2F*2Fwww.plosone.org*2Fstatic*2Fpolicies.action*23sharing%2F1%2F010f019bd54ab969-8a5b56c3-4c18-4591-97d9-b1160665c03c-000000%2Fi6IKbxAK_N9K10v0K2IXZ2KyyW6QzMxAd7mt_uJeJT8%3D246__%3BJSUlJSU!!H9nueQsQ!4fRBtrR60Fhs6cHkQ0QDjMizlLjJ5_tNmuHEFpx50KiI5xYImUBTtZMvF5O5_crPYXDkcw1wHol6cwBWZrQ%24&data=05%7C02%7Csomsuvro.basu%40p-95.com%7C7254a33ceb294ec61d7708de5834785c%7Ca3ca6855f73146e09a48a088d208b05e%7C1%7C0%7C639045179567929605%7CUnknown%7CTWFpbGZsb3d8eyJFbXB0eU1hcGkiOnRydWUsIlYiOiIwLjAuMDAwMCIsIlAiOiJXaW4zMiIsIkFOIjoiTWFpbCIsIldUIjoyfQ%3D%3D%7C0%7C%7C%7C&sdata=0T2h0B%2F3Te%2FkgTFmMo2JtgUfNbm4%2F4WumbSGfYrBpQA%3D&reserved=0) requires authors to make all data underlying the findings described in their manuscript fully available without restriction, with rare exception (please refer to the Data Availability Statement in the manuscript PDF file). The data should be provided as part of the manuscript or its supporting information, or deposited to a public repository. For example, in addition to summary statistics, the data points behind means, medians and variance measures should be available. If there are restrictions on publicly sharing data—e.g. participant privacy or use of data from a third party—those must be specified.

Reviewer #3: No

*Response: The Data Availability Statement has already been updated to explain why the datasets are not publicly available and how they can be requested. We share the Data Availability Statement, again below:*

***Data Availability Statement****: “The datasets generated during and/or analyzed during the current study are not publicly available. The data are owned by a third-party, Medical Data Vision (MDV) Co., Ltd, and the authors do not have permission to share the data. The data are available upon request to MDV (https://en.mdv.co.jp; contacts: Hiroyuki Tada [email: tada@mdv.co.jp] and Yuki Santo [email: santo_yuki@mdv.co.jp]).*

*The statistical codes are available upon request to the following contacts: statistics department, P95 (email: stat@p-95.com), and Hannah Volkman (Pfizer Inc; email: Hannah.Volkman@pfizer.com).”*

5. Is the manuscript presented in an intelligible fashion and written in standard English?

PLOS ONE does not copyedit accepted manuscripts, so the language in submitted articles must be clear, correct, and unambiguous. Any typographical or grammatical errors should be corrected at revision, so please note any specific errors here.

Reviewer #3: Yes

*Response: We thank Reviewer #3 for the confirmation.*

6. **Review Comments to the Author**

Please use the space provided to explain your answers to the questions above. You may also include additional comments for the author, including concerns about dual publication, research ethics, or publication ethics. (Please upload your review as an attachment if it exceeds 20,000 characters)

**Reviewer** **#3:**

- L344: The statement regarding the “nationally representative of DPC” listed as a study strength should be moderated. The analysis relies exclusively on MDV data, which represent only a subset of DPC hospitals. Moreover, the DPC system itself covers approximately 54% of hospital beds in Japan. “nationally representative” appear overstated and should be avoided or more carefully qualified.

*Response: We thank Reviewer #3 for the comment. We have adapted the sentence to take this into account. Please see L342-344 (in the revised clean manuscript): 'We used a database that includes DPC hospitalizations across different regions throughout Japan, which reduced the possibility of sampling bias.’*

- L204: The information concerning the total number of hospitalizations in Japan in 2017 does not appear essential (too broad) and could be omitted. The representativeness of the MDV data in relation to DPC hospitals and the national context is now sufficiently clarified in the Methods section.

*Response: The sentence L204 has been deleted.*

- L345-346: Presenting the six cardiovascular disease codes selected for this study as a strength should be moderated. Several comparable studies have included the full range of cardiovascular disease codes (I00–I99) (e.g., DOI: 10.1007/s40121-024-00951-0)

*Response: We thank Reviewer #3 for the comment. We have adapted the sentence to take this into account. Please see L344-346 (in the revised clean manuscript): ‘We also used an extensive list of ICD-10 codes, corresponding to broad definitions for respiratory outcomes and more specific for cardiovascular diseases, thereby improving the sensitivity of detecting RSV-related outcomes [16, 43-45].’*

*To support this amendment, we have added new references: nos. 43-45*

7. PLOS authors have the option to publish the peer review history of their article ([what does this mean?](https://eur02.safelinks.protection.outlook.com/?url=https%3A%2F%2Furldefense.com%2Fv3%2F__https%3A%2Ftrack.editorialmanager.com%2FCL0%2Fhttps%3A*2F*2Fjournals.plos.org*2Fplosone*2Fs*2Feditorial-and-peer-review-process*23loc-peer-review-history%2F1%2F010f019bd54ab969-8a5b56c3-4c18-4591-97d9-b1160665c03c-000000%2Fcq3nGFac781kEzyhP98_anW34ns5SzD0ne7JbpFDx5s%3D246__%3BJSUlJSUl!!H9nueQsQ!4fRBtrR60Fhs6cHkQ0QDjMizlLjJ5_tNmuHEFpx50KiI5xYImUBTtZMvF5O5_crPYXDkcw1wHol6HZJfGH4%24&data=05%7C02%7Csomsuvro.basu%40p-95.com%7C7254a33ceb294ec61d7708de5834785c%7Ca3ca6855f73146e09a48a088d208b05e%7C1%7C0%7C639045179567956450%7CUnknown%7CTWFpbGZsb3d8eyJFbXB0eU1hcGkiOnRydWUsIlYiOiIwLjAuMDAwMCIsIlAiOiJXaW4zMiIsIkFOIjoiTWFpbCIsIldUIjoyfQ%3D%3D%7C0%7C%7C%7C&sdata=%2FwW5PEV%2FUY1LH6uWHHtgqYs%2FfNslZ7GycBwu6BhEL8s%3D&reserved=0)). If published, this will include your full peer review and any attached files.

*Response: We are fine to publish the peer review history of our article.*

If you choose “no”, your identity will remain anonymous but your review may still be made public.


**Do you want your identity to be public for this peer review?** For information about this choice, including consent withdrawal, please see our [Privacy Policy](https://eur02.safelinks.protection.outlook.com/?url=https%3A%2F%2Furldefense.com%2Fv3%2F__https%3A%2Ftrack.editorialmanager.com%2FCL0%2Fhttps%3A*2F*2Fwww.plos.org*2Fprivacy-policy%2F1%2F010f019bd54ab969-8a5b56c3-4c18-4591-97d9-b1160665c03c-000000%2FdJWrRFyQbm39bsc_wjvBPxfg3-lnS72UOfMbTgQ5kd8%3D246__%3BJSUl!!H9nueQsQ!4fRBtrR60Fhs6cHkQ0QDjMizlLjJ5_tNmuHEFpx50KiI5xYImUBTtZMvF5O5_crPYXDkcw1wHol6WPE-F6g%24&data=05%7C02%7Csomsuvro.basu%40p-95.com%7C7254a33ceb294ec61d7708de5834785c%7Ca3ca6855f73146e09a48a088d208b05e%7C1%7C0%7C639045179567978758%7CUnknown%7CTWFpbGZsb3d8eyJFbXB0eU1hcGkiOnRydWUsIlYiOiIwLjAuMDAwMCIsIlAiOiJXaW4zMiIsIkFOIjoiTWFpbCIsIldUIjoyfQ%3D%3D%7C0%7C%7C%7C&sdata=l4Vcp9N1JUya3npbdWgXTaQGtTM5e57Ydmq7OWjB4NM%3D&reserved=0).

Reviewer #3: No
